# Estimation of Time-Varying, Intrinsic and Reflex Dynamic Joint Stiffness during Movement. Application to the Ankle Joint

**DOI:** 10.3389/fncom.2017.00051

**Published:** 2017-06-09

**Authors:** Diego L. Guarín, Robert E. Kearney

**Affiliations:** Biomedical Engineering Department, McGill UniversityMontréal, QC, Canada

**Keywords:** biological system modeling, nonlinear system identification, time-varying systems, dynamic joint stiffness, joint neuromechanics

## Abstract

Dynamic joint stiffness determines the relation between joint position and torque, and plays a vital role in the control of posture and movement. Dynamic joint stiffness can be quantified during quasi-stationary conditions using disturbance experiments, where small position perturbations are applied to the joint and the torque response is recorded. Dynamic joint stiffness is composed of intrinsic and reflex mechanisms that act and change together, so that nonlinear, mathematical models and specialized system identification techniques are necessary to estimate their relative contributions to overall joint stiffness. Quasi-stationary experiments have demonstrated that dynamic joint stiffness is heavily modulated by joint position and voluntary torque. Consequently, during movement, when joint position and torque change rapidly, dynamic joint stiffness will be Time-Varying (TV). This paper introduces a new method to quantify the TV intrinsic and reflex components of dynamic joint stiffness during movement. The algorithm combines ensemble and deterministic approaches for estimation of TV systems; and uses a TV, parallel-cascade, nonlinear system identification technique to separate overall dynamic joint stiffness into intrinsic and reflex components from position and torque records. Simulation studies of a stiffness model, whose parameters varied with time as is expected during walking, demonstrated that the new algorithm accurately tracked the changes in dynamic joint stiffness using as little as 40 gait cycles. The method was also used to estimate the intrinsic and reflex dynamic ankle stiffness from an experiment with a healthy subject during which ankle movements were imposed while the subject maintained a constant muscle contraction. The method identified TV stiffness model parameters that predicted the measured torque very well, accounting for more than 95% of its variance. Moreover, both intrinsic and reflex dynamic stiffness were heavily modulated through the movement in a manner that could not be predicted from quasi-stationary experiments. The new method provides the tool needed to explore the role of dynamic stiffness in the control of movement.

## 1. Introduction

The role of the short-latency stretch reflex during movement remains controversial (Dietz et al., 1979; Sinkjaer et al., 1996; Zehr and Stein, 1999). While some studies suggest that reflex response serves to facilitate all voluntary movements (Dufresne et al., 1980; Gottlieb and Agarwal, 1980), others have proposed that the reflex response plays a role only in extreme or pathological cases (Dietz et al., 1980), or during early adaptation to new tasks or conditions (Burdet et al., 2013).

EMG is often used to study the functional role of reflexes (Dietz et al., 1979; Stein and Capaday, 1988; Zehr and Stein, 1999; Burdet et al., 2013). However, EMG is influenced by factors other than reflexes, such as voluntary activity, and it is difficult to separate the reflex EMG response from the overall EMG activity. In addition, the relation between EMG and joint torque is influenced by muscle length and contraction velocity, so that is difficult to estimate the mechanical contributions of stretch reflex from EMG alone (Toft et al., 1991; Stein and Kearney, 1995; Kearney et al., 1999).

H-reflexes have also been used to quantify the reflex activity (Sinkjaer et al., 1993). However, H-reflexes bypass the response of muscle spindles to joint position changes, which can be heavily modulated during function via γ-motor neurons (Sinkjaer et al., 1996). In addition, direct stimulation of the nerve may excite a range of afferent mechanisms that project to α−motorneurons (e.g., skin sensors, Golgi tendon organs) so that the resultant response will be generated by unphysiological combination of afferent activity (Van der Helm et al., 2002). Consequently, the functional relevance of these H-reflex studies is not completely clear.

A better approach would be to directly measure the mechanical consequences of reflex activity. However, it is difficult to separate reflex torques from those due to the mechanical or intrinsic properties of the muscle and connective tissue. Experimentally this has been achieved by comparing the mechanical behavior of a joint before and after deafferentation using surgery (Kirsch et al., 1994), or some other manipulation to suppress the reflex response (Dietz et al., 1980; Allum and Mauritz, 1984). However, it is not possible to be sure that the deafferentation process affects only the stretch reflex, and to what extent. This process will likely also affect the intrinsic properties of the joint (Kearney et al., 1997; Van der Helm et al., 2002).

An alternative approach is to perform the “*deafferentation*” by using mathematical models and system identification techniques to separate the mechanical effects of intrinsic and reflex mechanisms. System identification techniques, using small, random position or torque perturbations to excite the intrinsic and reflex dynamics, have been successfully applied to multiple joints with different model types (Gottlieb and Agarwal, 1978; Zhang and Rymer, 1997; Van der Helm et al., 2002; Klomp et al., 2014). These models have typically been linear; however, the mechanical response produced by stretch reflexes are highly nonlinear (Stein and Kearney, 1995), so that these models fail to completely characterize the stretch reflex mechanisms or simply ignore it. The parallel-cascade model, proposed by Kearney et al. (1997), describes the intrinsic and stretch reflex mechanisms in terms of dynamic joint stiffness, that determines the dynamic relation between joint position and torque. Intrinsic dynamic stiffness, also referred to as joint impedance, arises from the inertial and visco-elastic properties of the joint, passive tissue, and active muscle fibers, and is described by a linear model relating joint position and torque. Reflex dynamic stiffness arises from changes in muscle activation due to the short-latency stretch reflex, and is described by a nonlinear, Hammerstein model relating joint velocity and torque (Kearney and Hunter, 1990).

Successful applications of these analytical techniques have been typically limited to stationary conditions, where the dynamic properties of the joint remain constant for the duration of the experiment. Such experiments have shown that the parallel-cascade model parameters change with joint position and voluntary torque (Mirbagheri et al., 2000; Guarin et al., 2013). Consequently, during most functional activities when the joint position and voluntary torque change rapidly and continuously, the dynamic joint stiffness model parameters will be time-varying (TV).

Several studies have characterized dynamic joint stiffness during TV conditions by modeling the intrinsic and reflex response together using a single linear model (Bennett et al., 1992; MacNeil et al., 1992; Kirsch and Kearney, 1997; Rouse et al., 2014; Lee and Hogan, 2015). These type of models cannot provide any information regarding the modulation of reflex mechanisms and likely overestimate the contribution of intrinsic mechanisms to the overall dynamic joint stiffness. We have introduced methods to estimate intrinsic and stretch reflex mechanisms using the parallel-cascade model structure during TV conditions; however, these methods require very large data sets for parameter estimation, which severely limits their application (Giesbrecht et al., 2006; Ludvig et al., 2011; Guarin and Kearney, 2012, 2015b); or make the strong assumption that there is a static-nonlinear relation between the parallel-cascade model parameters and joint position or torque (Sobhani Tehrani et al., 2013; Jalaleddini et al., 2015). Despite their limitations, these studies have shown that the interpolation of parameter values obtained from stationary experiments does not describe dynamic joint stiffness during TV conditions. Therefore, methods able to track the fast, large changes in the model parameters using short data records are required to characterize the modulation of the dynamic joint stiffness during function.

This paper develops and validates a novel method to estimate the intrinsic and reflex components of dynamic joint stiffness during periodic movements. This method improves over previous algorithms in several ways: (i) it reduces the size of the data set required for accurate parameter estimation; and (ii) it parametrizes the system and noise plants independently, which eliminates biases in parameter estimates due to the colored noise present in measurements of joint torque.

This paper is organized as follows: Section 2 presents the TV, parallel-cascade model of dynamic joint stiffness and introduces a novel re-parameterization that approximates, the non-linear, TV model with a set of linear, time-invariant models. It then introduces an algorithm to estimate the parameters of this model using data acquired during periodic, TV conditions. Section 3 describes a simulation study that evaluated the performance of the new model parameterization and identification algorithm. Section 4 demonstrates the practical application of the algorithm by using it to estimate intrinsic and reflex dynamic ankle stiffness during experiments where movements were imposed on subjects while they exerted a constant voluntary torque. Section 5 summarizes the contributions and discusses some important aspects underlying the method and its application.

## 2. Model formulation and parameter identification

### 2.1. Joint position perturbations and torque

Estimation of dynamic joint stiffness requires the application of small position perturbations that do not modify joint position and have power over a wide enough range of frequencies to excite the system adequately (Kearney and Hunter, 1990). Consequently, to estimate dynamic joint stiffness during movement, small position perturbations must be superimposed on the movement trajectory, producing an overall, perturbed joint position given by

(1) $$\theta (t_{k})=\theta_{0}(t_{k})+\theta_{p}(t_{k}),$$

where θ_0_(*t*_*k*_) is the movement trajectory and θ_*p*_(*t*_*k*_) is the position perturbation.

Under stationary conditions, when the joint trajectory and voluntary torque are almost constant, the net moment at the joint is

$$TQ(t_{k})=TQ_{0}+TQ_{p}(t_{k}),$$

where *TQ*_0_ is a constant torque, produced by passive mechanisms due to θ_0_ (which might be equal to zero if the joint is at its neutral position), and by active mechanisms due to the constant muscle activation; and *TQ*_*p*_(*t*_*k*_) is a perturbation torque, produced by the excitation of intrinsic and reflex mechanisms given by

$$TQ_{p}(t_{k})=TQ_{I}(t_{k})+TQ_{R}(t_{k})$$

where *TQ*_*I*_(*t*_*k*_) and *TQ*_*R*_(*t*_*k*_) are the torques produced by the intrinsic and reflex mechanisms, which cannot be measured directly. Under stationary conditions, an estimate of the perturbation torque can be retrieved from measurements of total joint torque by removing the constant offset *TQ*_0_. The perturbation position and torque can then be used to estimate the intrinsic and reflex contributions to the total torque.

In contrast, under TV conditions, when the joint trajectory (θ_0_(*t*_*k*_)) and/or the muscle activation level vary, the torque produced by passive and voluntary mechanisms (*TQ*_0_(*t*_*k*_)), is no longer constant. Consequently, estimating the perturbation torque from measurements of total joint torque requires three steps: First, a perturbed joint trajectory is applied and the total joint torque, given by

$$TQ(t_{k})=TQ_{0}(t_{k})+TQ_{p}(t_{k}),$$

is recorded. Second, an unperturbed joint trajectory is applied and the joint torque $TQ_{0}^{*}\left(t_{k}\right)$ is recorded. Finally, the difference between the net joint torque in the two experiments is computed to estimate the torque due to the perturbations. However, it is not realistic to expect that the joint will follow exactly the same trajectory and/or that the subject will exert exactly the same voluntary torque in the perturbed and unperturbed experiments. Therefore, under TV conditions, the perturbation torque will be given by

(2) $$TQ_{p}(t_{k})=TQ_{I}(t_{k})+TQ_{R}(t_{k})+TQ_{\Delta }(t_{k}),$$

where *TQ*_Δ_(*t*_*k*_) is an additional torque due to difference in passive and voluntary torques during the perturbed and unperturbed experiments.

### 2.2. Time-varying dynamic joint stiffness

Once the perturbation position and torque are available, system identification can be used to separate the intrinsic and reflex components analytically. Under stationary conditions, this can be achieved by modeling the overall dynamic joint stiffness with a parallel-cascade model, where intrinsic stiffness is described by a linear system relating joint position and intrinsic torque, and reflex stiffness by a Hammerstein system relating joint velocity and reflex torque (Kearney et al., 1997; Guarin et al., 2013; Jalaleddini et al., 2016).

**Figure 1:**
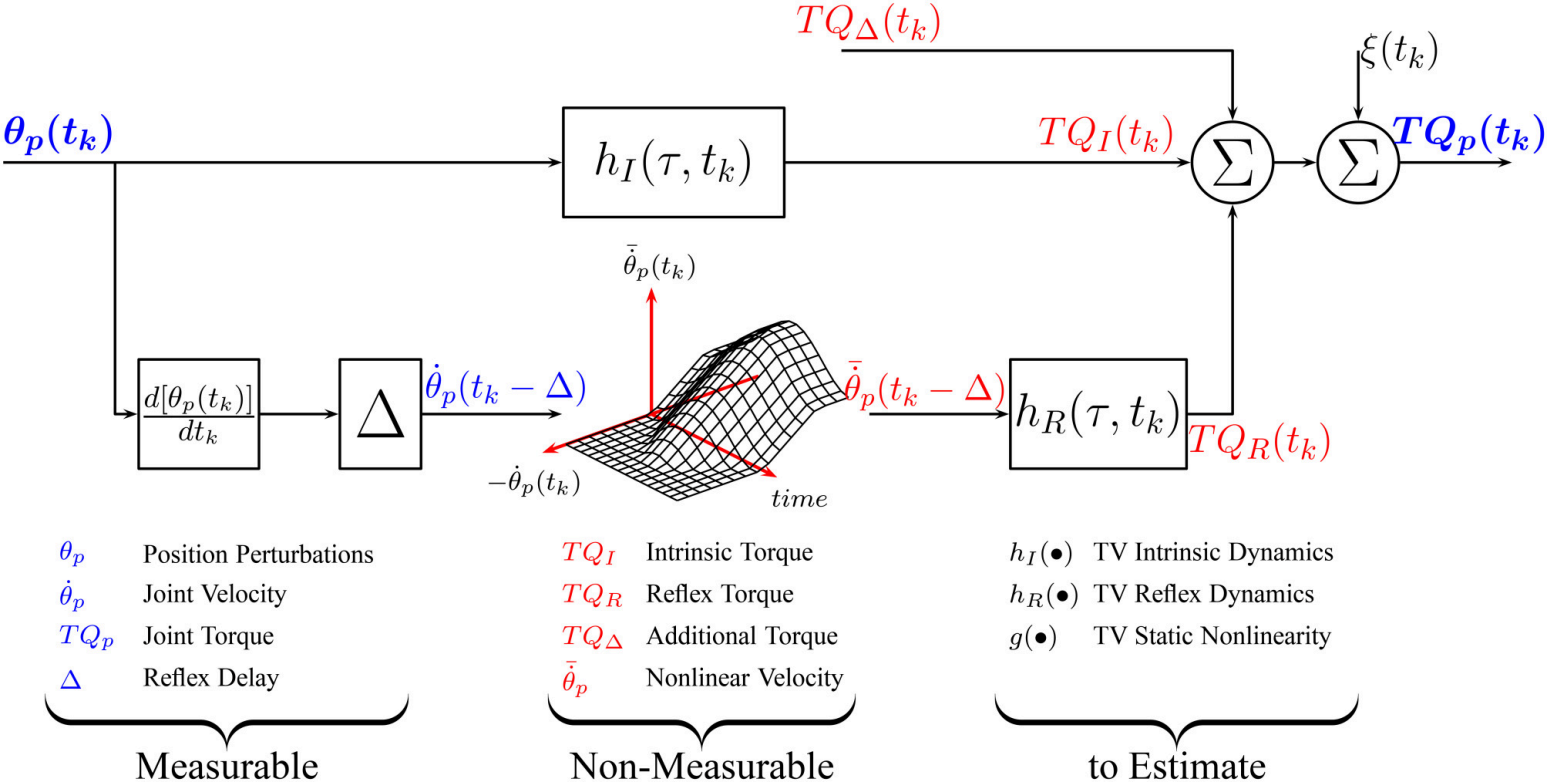
Time-Varying, Parallel-Cascade model structure representing the intrinsic and reflex responses to small position perturbations. Measurable signals are shown in blue while those that can only be estimated are shown in red.

Under TV conditions, a TV version of the parallel-cascade structure, shown in Figure 1, has been successfully applied to describe the overall dynamic joint stiffness (Giesbrecht et al., 2006; Ludvig et al., 2011; Guarin and Kearney, 2012, 2015b; Jalaleddini et al., 2017). However, the identification algorithms used to estimated the TV model parameters require very large data sets and so are difficult to use in practice.

Here, we introduce an alternative parameterization of the TV, nonlinear, parallel-cascade model of dynamic joint stiffness that transforms it into a set of pseudo-linear, time-invariant models. Next, we will introduce an identification algorithm that uses a small data set to estimate the TV model parameters.

#### 2.2.1. Intrinsic dynamic stiffness

Intrinsic dynamic stiffness is usually described by a second order, linear model with limb inertia, joint viscosity, and static stiffness relating perturbation position and torque (Kearney and Hunter, 1990)

(3) $$TQ_{I}(t_{k})=K(t_{k})\theta_{p}(t_{k})+B(t_{k})\frac{d[\theta_{p}(t_{k})]}{dt_{k}}+I\frac{d^{2}[\theta_{p}(t_{k})]}{d^{2}t_{k}},$$

where *K*(*t*_*k*_), *B*(*t*_*k*_) and *I* are the intrinsic static stiffness, viscosity and inertia. However, recent experimental evidence suggests that the intrinsic dynamics stiffness is more complex than second-order (Sobhani Tehrani et al., 2017). Therefore, we choose to describe intrinsic stiffness with the TV, non-parametric model

(4) $$TQ_{I}(t_{k})=\sum_{\tau =-L}^{\tau =L}h_{I}(\tau ,t_{k})\theta_{p}(t_{k}-\tau ),$$

where *h*_*I*_(τ, *t*_*k*_) is a TV, impulse response function (IRF) that requires no a priori assumption of model order. The length of the system memory must be specified *a priori*, and there is much evidence than a memory of 40 *ms* is adequate for the ankle joint (Kearney et al., 1997). Therefore, intrinsic dynamic stiffness is represented by a two sided IRF with a memory from −40 *ms* to 40 *ms*.

##### 2.2.1.1. Model re-parameterization

The TV parameters in Equation (4) will be approximated by a linear combination of basis functions as

$$h_{I}\left(\tau ,t_{k}\right)=\sum_{j\;=\;0}^{j\;=\;n_{\lambda }}\lambda_{\tau ,j}\Lambda_{j}\left(t_{k}\right),$$

where ${\left\{\Lambda_{j}\left(t_{k}\right)\right\}}_{j=0}^{j=n_{\lambda }}$ are a set of time-varying basis functions and λ_τ, *j*_ their coefficients. Intrinsic dynamic stiffness can then be approximated by the linear, time-invariant (LTI) model

(5) $$TQ_{I}(t_{k})=\sum_{\tau =-L}^{\tau =L}\sum_{j=0}^{j=n_{\lambda }}\lambda_{\tau ,j}\Lambda_{j}\left(t_{k}\right)\theta_{p}(t_{k}-\tau ).$$

#### 2.2.2. Reflex dynamic stiffness

Reflex dynamic stiffness can be described by a series connection of a differentiator, a delay of 40 *ms* and a Hammerstein system, comprising the series combination of a static-nonlinearity and a second-order, linear dynamic system, relating joint velocity and reflex torque (Kearney et al., 1997; Guarin et al., 2013; Guarin and Kearney, 2015b). The input-output relation is given by

(6a) $${\bar{\dot{\theta }}}_{p}(t_{k})=g\left({\dot{\theta }}_{p}(t_{k}),t_{k}\right),$$

(6b) $$\begin{array}{l} \frac{d^{2}[TQ_{R}(t_{k})]}{dt_{k}^{2}}+2\zeta (t_{k})\omega (t_{k})\frac{d[TQ_{R}(t_{k})]}{dt_{k}}+\omega^{2}(t_{k})TQ_{R}(t_{k})\\ \;=G(t_{k})\omega^{2}(t_{k}){\bar{\dot{\theta }}}_{p}(t_{k}), \end{array}$$

where ${\overset{\cdot }{\theta }}_{p}\left(t_{k}\right)$ is the delayed joint velocity, and *g*(•, *t*_*k*_) is a TV, static non-linearity. *G*(*t*_*k*_), ω(*t*_*k*_), and ζ(*t*_*k*_) are the gain, natural frequency and damping of the reflex linear dynamics.

This TV, continuous-time model can be approximated by the set of discrete-time, transfer function models

(7) $$TQ_{R}(t_{k})=\frac{b_{0}(t_{k})\left(1+2q^{-1}+q^{-1}\right)}{1+a_{1}(t_{k})q^{-1}+a_{2}(t_{k})q^{-2}}{\bar{\dot{\theta }}}_{p}(t_{k}),$$

where *b*_0_(*t*_*k*_), *a*_1_(*t*_*k*_) and *a*_2_(*t*_*k*_) are discrete-time, TV parameters and *q*^−1^ is the backward shift operator. The continuous-time and discrete-time parameters are related to each other by

$$\begin{array}{l} G(t_{k})=4\left[\frac{b_{0}(t_{k})}{1+a_{1}(t_{k})+a_{2}(t_{k})}\right],\\ \omega (t_{k})=\frac{2}{T_{s}}{\left[\frac{1+a_{1}(t_{k})+a_{2}(t_{k})}{1-a_{1}(t_{k})+a_{2}(t_{k})}\right]}^{1/2},\\ \zeta (t_{k})=\frac{1-a_{2}(t_{k})}{{\left[\left(1+a_{1}(t_{k})+a_{2}(t_{k})\right)\left(1-a_{1}(t_{k})+a_{2}(t_{k})\right)\right]}^{1/2}}. \end{array}$$

where *T*_*s*_ is the sampling time in seconds.

##### 2.2.2.1. Model re-parameterization

The TV, static non-linearity will be approximated by

$${\bar{\dot{\theta }}}_{p}\left(t_{k}\right)=g\left({\dot{\theta }}_{p}(t_{k}),t_{k}\right)\approx \sum_{i=0}^{i=n_{c}}c_{i}\left(t_{k}\right)C_{i}\left({\dot{\theta }}_{p}\left(t_{k}\right)\right),$$

where *C*_*i*_(•) are a set of pre-defined basis functions (e.g., polynomials, radial basis) and *c*_*i*_ are their TV coefficients. Following the same procedure as before, these are approximated by a linear combination of basis functions as

$$c_{i}(t_{k})=\sum_{j=0}^{j=n_{\gamma }}\gamma_{i,j}\Gamma_{j}\left(t_{k}\right),$$

where ${\left\{\Gamma_{j}\left(t_{k}\right)\right\}}_{j=0}^{j=n_{\gamma }}$ are a set of time-varying basis functions and γ_*i, j*_ their coefficients.

Similarly, the TV parameters of the linear dynamic element will be approximated by a linear combination of basis functions

$$\begin{array}{l} b_{0}\left(t_{k}\right)=\sum_{j=0}^{j=n_{\beta }}\beta_{0,j}\Psi_{j}\left(t_{k}\right),\\ a_{i}\left(t_{k}\right)=\alpha_{i,0}+\sum_{j=1}^{j=n_{\alpha }}\alpha_{i,j}\Pi_{j}\left(t_{k}\right),\;i=0,\ldots ,n_{a}. \end{array}$$

where α_*i*,0_ ≠ 0; ${\left\{\Psi_{j}\left(t_{k}\right)\right\}}_{j=0}^{j=n_{\beta }}$ and ${\left\{\Pi_{j}\left(t_{k}\right)\right\}}_{j=0}^{j=n_{\alpha }}$ are sets of time-varying basis functions with Π_0_(*t*_*k*_) = 1, ∀*t*_*k*_; β_0, *j*_, and α_*i, j*_ their coefficients.

Using the approximations with the basis functions, the relation between the reflex torque and joint velocity is now time-invariant and can be described by the discrete-time, time-invariant, Hammerstein system

(8a) $${\bar{\dot{\theta }}}_{p}\left(t_{k}\right)=\;\sum_{i=0}^{i=n_{c}}\sum_{j=0}^{j=n_{\gamma }}\gamma_{i,j}\Gamma_{j}\left(t_{k}\right)C_{i}\left({\dot{\theta }}_{p}\left(t_{k}\right)\right),$$

(8b) $$\begin{array}{l} TQ_{R}\left(t_{k}\right)=\frac{1}{F\left(q^{-1}\right)}[-\sum_{j\;=\;1}^{n_{\alpha }}\alpha_{1,j}\Pi_{j}\left(t_{k}\right)TQ_{R}\left(t_{k}-1\right)\\ -\sum_{j\;=\;1}^{n_{\alpha }}\alpha_{2,j}\Pi_{j}\left(t_{k}\right)TQ_{R}\left(t_{k}-2\right)+\sum_{j\;=\;0}^{j=n_{\beta }}\beta_{0,j}\Psi_{j}\left(t_{k}\right){\bar{\dot{\theta }}}_{p}\left(t_{k}\right)], \end{array}$$

where *F*(*q*^−1^) is the polynomial

$$F\left(q^{-1}\right)=1+\alpha_{1,0}q^{-1}+\alpha_{2,0}q^{-2},$$

#### 2.2.3. Other components

*TQ*_Δ_(*t*_*k*_) is expected to be a stochastic, low-frequency signal that will be described by a linear combination of basis functions

(9) $$TQ_{\Delta }(t_{k})=\sum_{j=0}^{j=n_{p}}p_{i}P_{i}(t_{k}),$$

where ${\left\{P_{i}\left(t_{k}\right)\right\}}_{j=0}^{j=n_{p}}$ are a set of time-varying basis functions and *p*_*i*_ their coefficients.

#### 2.2.4. Overall joint stiffness

Using these re-parameterizations, the overall relation between joint position and torque, shown in Figure 1, can be approximated by the LTI models shown in Equations (5), (8a), (8b), and (9) the unknown parameters

(10a) $$\rho_{I}=\left[\lambda_{-L,0}\cdots \lambda_{-L,n_{\lambda }}\cdots \lambda_{L,0}\cdots \lambda_{L,n_{\lambda }}\right],$$

(10b) $$\begin{array}{l} \rho_{R}=[\alpha_{1,0}\cdots \alpha_{1,n_{\alpha }}\cdots \alpha_{2,0}\cdots \alpha_{2,n_{\alpha }}\;\beta_{0,0}\cdots \beta_{0,n_{\beta }}\\ \;\gamma_{0,0}\cdots \gamma_{0,n_{\gamma }}\cdots \gamma_{n_{c},0}\cdots \gamma_{n_{c},n_{\gamma }}], \end{array}$$

(10c) $$\rho_{\Delta }=\left[p_{0}\cdots p_{n_{p}}\right],$$

where **ρ**_*I*_, **ρ**_*R*_, and **ρ**_Δ_ are vectors containing the unknown parameters used to describe the intrinsic, reflex and additional torques, respectively.

### 2.3. Identification of TV, dynamic joint stiffness

We now describe an algorithm for the identification of the re-parametrized models of *TQ*_*I*_(*t*_*k*_), *TQ*_*R*_(*t*_*k*_), and *TQ*_Δ_(*t*_*k*_). There are four key elements to the algorithm: First, as Figure 1 illustrates, these torques cannot be measured directly so the models describing each component cannot be estimated directly from measured data. Consequently, the intrinsic and reflex components will be estimated using an iterative algorithm that estimates the parameters of each pathway sequentially, removing the influence of the other pathways in the total torque before estimating the parameters of each component (Kearney et al., 1997; Guarin and Kearney, 2015b).

Second, the parameters of each element of the Hammerstein system that represents the reflex component will be estimated using a second iterative algorithm. This method estimates the coefficients of the static nonlinearity and reflex dynamics iteratively using a coordinate ascent approach. The algorithm is guaranteed to converge to the true values under general conditions (Bai and Li, 2004; Guarin and Kearney, 2015a).

Third, an instrumental variable approach, that provides unbiased estimates of the model parameters even in the presence of non-white noise, will be used to estimate the reflex linear dynamic element (Laurain et al., 2010; Guarin and Kearney, 2016).

Finally, the identification algorithm assumes that there are available multiple input-output trials presenting the same time-varying behavior. The algorithm exploits this to estimate the parameters' time-course from multiple realization of input-output data. Moreover, the algorithm assumes that the time-varying behavior is periodic and it automatically estimates the initial conditions at each trial.

The identification algorithm combines two TV identification methodologies: temporal expansion and ensemble approaches. We recently introduced this hybrid identification approach and showed that it can track faster parameters changes than the temporal expansion method while requiring less data than classical ensemble approaches (Guarin and Kearney, 2016).

#### 2.3.1. Identification algorithm

Assume that *n* cycles, each with *N* data points, of joint position and torque were measured for both the unperturbed and perturbed joint movements. The position perturbation and torque signals are computed by aligning and subtracting the unperturbed from the perturbed measurements. Following Equation (2), the noise-free perturbation torque for *n* cycles can be organized in matrix form as

(11) $$\left[\begin{array}{c} TQ_{p}\{1\}\\ \vdots \\ TQ_{p}\{n\} \end{array}\right]=\left[\begin{array}{c} TQ_{I}\{1\}\\ \vdots \\ TQ_{I}\{n\} \end{array}\right]+\left[\begin{array}{c} TQ_{R}\{1\}\\ \vdots \\ TQ_{R}\{n\} \end{array}\right]+\left[\begin{array}{c} TQ_{\Delta }\{1\}\\ \vdots \\ TQ_{\Delta }\{n\} \end{array}\right],$$

where

$$TQ_{p}\{j\}={\left[TQ_{p}(1)\{j\}\cdots TQ_{p}(N)\{j\}\right]}^{T},$$

is the perturbation torque for the j-th cycle. The identification algorithm assumes that intrinsic and reflex dynamics have the same TV behavior in each cycle and that the TV model parameters are periodic. In contrast, *TQ*_Δ_(*t*_*k*_) is assumed to be different for each cycle, so that the parameters describing it are different for each cycle.

The identification algorithm proceeds as follows:

1. Initialize
  $${\hat{TQ}}_{I}\{j\}={\hat{TQ}}_{R}\{j\}=O,\;j=1,\cdots ,n.$$
2. Estimate *TQ*_Δ_ for each cycles as
  $${\tilde{TQ}}_{\Delta }\{j\}=TQ_{p}\{j\}-\left({\hat{TQ}}_{I}\{j\}+{\hat{TQ}}_{R}\{j\}\right)$$
  - Use ${\tilde{TQ}}_{\Delta }\left\{j\right\}$ and the linear, identification algorithm introduced in Guarin and Kearney (Submitted) to estimate ${\hat{\rho }}_{\Delta }$ for each cycle.
  - Use these estimates to predict ${\hat{TQ}}_{\Delta }\{j\}$ for each cycle.
3. Estimate the intrinsic torque as
  $${\tilde{TQ}}_{I}\{j\}=TQ_{p}\{j\}-\left({\hat{TQ}}_{R}\{j\}+{\hat{TQ}}_{\Delta }\{j\}\right)$$
  - Use the current prediction of the intrinsic torque and the perturbation position with the algorithm introduced in Guarin and Kearney (Submitted) to estimate ${\hat{\rho }}_{\Delta }$. As the joint trajectory is the same at each realization in the ensemble, the algorithm estimates a single set of coefficients using all the realizations.
  - Use these estimates and the perturbation position to update the prediction of ${\hat{TQ}}_{I}\left\{j\right\}$ for each cycle.
4. Estimate the reflex torque as
  $${\tilde{TQ}}_{R}\{j\}=TQ_{p}\{j\}-\left({\hat{TQ}}_{I}\{j\}+{\hat{TQ}}_{\Delta }\{j\}\right)$$
  - Use current prediction of the reflex torque, the perturbation velocity and the algorithm introduced in Guarin and Kearney (2015a) to estimate ${\hat{\rho }}_{R}$. As the joint trajectory is the same at each realization in the ensemble, the algorithm estimates a single set of coefficients using all the realizations.
  - Use these estimates and the perturbation velocity to update the prediction of ${\hat{TQ}}_{R}\left\{j\right\}$ for each cycle.
5. Compute the net predicted torque for all cycles as
  $${\hat{TQ}}_{p}\{j\}={\hat{TQ}}_{I}\{j\}+{\hat{TQ}}_{R}\{j\}+{\hat{TQ}}_{\Delta }\{j\}$$
  and calculate the variance accounted for (*%VAF*) between the predicted and measured torque signals as
  $$\%VAF=\left[1-\frac{\sum_{t_{k}=1}^{t_{k}=N*n}{\left(TQ_{p}(t_{k})-{\hat{TQ}}_{p}(t_{k})\right)}^{2}}{\sum_{t_{k}=1}^{t_{k}=N*n}{\left(TQ_{p}(t_{k})\right)}^{2}}\right]\times 100\%,$$
  where N^*^n is the total number of samples.
6. Repeat the procedure from step 2 until successive iterations fail to improve the *%VAF*.

The identification algorithm predicts the intrinsic (${\hat{TQ}}_{I}\left(t_{k}\right)$), reflex (${\hat{TQ}}_{R}\left(t_{k}\right)$), and additional (${\hat{TQ}}_{\Delta }\left(t_{k}\right)$) torques, as well as the model parameters ${\hat{\rho }}_{I}$ and ${\hat{\rho }}_{R}$. A Matlab implementation of this algorithm and an application example are provided by DLG in GitHub^1^

## 3. Simulation study

### 3.1. Methods

The accuracy of the new algorithm was evaluated using simulations of TV, dynamic ankle stiffness throughout a periodic movement resembling the ankle movement during gait.

#### 3.1.1. Simulated model

**Figure 2:**
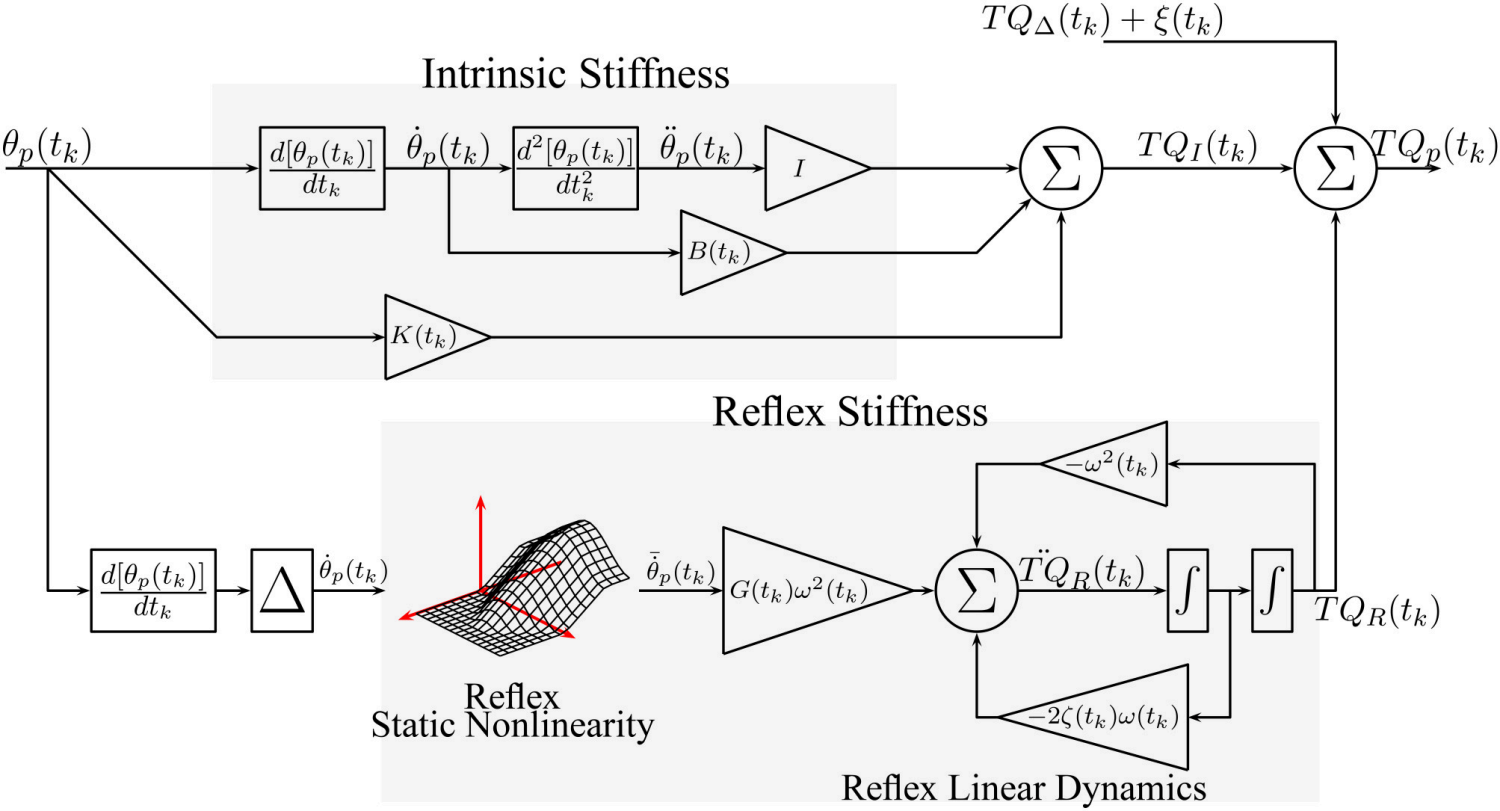
Simulated, TV, Parallel-Cascade model. Intrinsic dynamic stiffness was modeled as a TV, second order, continuous-time system. Reflex dynamic stiffness was modeled as a Hammerstein system comprising a TV static-nonlinearity followed by a second order, continuous-time system.

Figure 2 shows the TV, dynamic joint stiffness model used in the simulation. Intrinsic stiffness was simulated as a TV, continuous-time, second-order system. Reflex stiffness was modeled as the series connection of a 40 ms delay, a differentiator, and a Hammerstein system whose static-nonlinear element was a half-wave rectifier with a TV threshold (*th*(*t*_*k*_)), and whose linear dynamic element was a TV, continuous-time, second-order system. The model was simulated in Simulink (the MathWorks) using a third order solver with a sampling rate of 1 kHz. Each simulated cycle lasted 1.4 s, which is equivalent to slow walking Sinkjaer et al. (1996); 40 cycles were simulated so that each trial lasted for 56 s. Perturbation position and torque were filtered and decimated to 100 Hz for analysis. The 56 s trial was repeated 100 times with a different input and noise realizations to compute statistical properties for the parameter estimates.

##### 3.1.1.1. Model parameters

**Figure 3:**
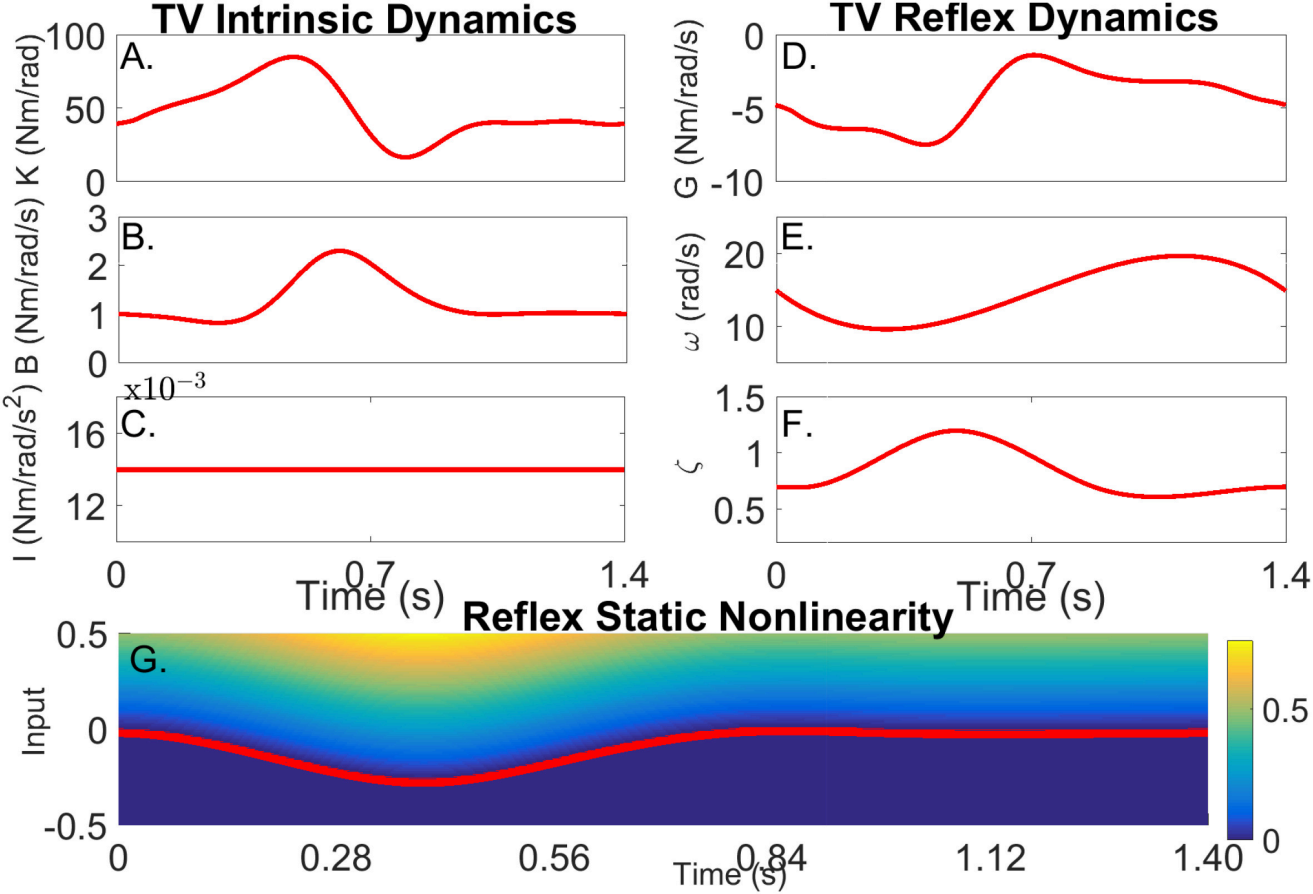
Simulated time-varying model parameters as a function of time. **(A)** Intrinsic Static Stiffness (*K*), **(B)** viscosity (*B*), and **(C)** inertia (*I*). **(D)** Reflex gain (*G*), **(E)** natural frequency (ω), **(F)** damping (ζ), and **(G)** reflex static-nonlinearity with the red line showing the TV threshold.

Figure 3 shows how the simulated parameters were varied periodically in the simulations. The variation of the intrinsic stiffness parameters, shown in Figures 3A–C, was based on results reported by Lee et al. (2016).

The variations of the parameters of the linear, reflex dynamics are shown in Figures 3D–F. The reflex gain changes were based on those reported by Sinkjaer et al. (1996), while those of the natural frequency and damping were generated by interpolating results from stationary experiments at different joint positions (Guarin et al., 2013). The threshold of the reflex static nonlinearity changed during the portion of the cycle where the reflex gain was largest, and remained constant at zero during the remainder of the cycle.

#### 3.1.2. Typical trial

##### 3.1.2.1. Input

**Figure 4:**
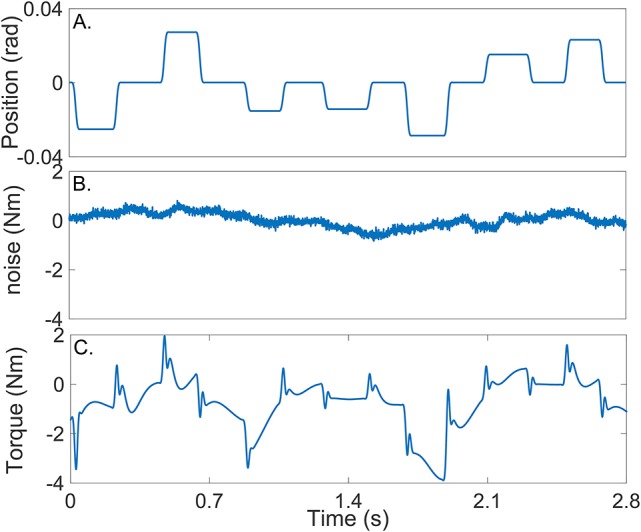
Typical simulation results: **(A)** position perturbations (input), **(B)** experimental noise and **(C)** perturbation torque (output).

Figure 4A shows the position input perturbation sequence which was a *Pseudo Random Arbitrary Level Distributed Signal* (PRALDS) with a random switching rate drawn from a uniform distribution between 250 and 350 *ms*, and a peak-to-peak amplitude of 0.06 rad. PRALDS signals have velocities distributed over the entire range of possible values and so it provides a rich set of values with which to estimate the reflex static-nonlinearity (Jalaleddini and Kearney, 2013).

##### 3.1.2.2. Experimental noise

Figure 4B shows a realization of the noise used in the simulations. This was obtained from a library of ankle torque records acquired while subjects maintained a constant torque at a fixed ankle position (Ranjbaran et al., 2013). The library comprised 100 records each lasting 60 s, from six subjects generating dorsiflexing torques corresponding to 5, 10, and 15% of their maximum voluntary torque. The experimental noise signal is composed of a low-frequency trend (corresponding to *TQ*_Δ_(*t*_*k*_)), physiological tremor, 60 Hz noise, and white-Gaussian measurement noise (Bezrukov et al., 2003; Ranjbaran et al., 2013). For each simulation trial, a 56 s section of the recorded torque noise was chosen at random from the library, its mean removed, and its amplitude adjusted to give an average signal-to-noise ratio (SNR) of 15 dB across the trial. This SNR is lower than that expected experimentally; (Ludvig and Kearney, 2007) reported it to be around 40 dB.

##### 3.1.2.3. Output

Figure 4C shows the noise-free output-torque, the sum of the simulated intrinsic and reflex torques.

#### 3.1.3. Basis functions

Cubic B-splines were selected as the basis functions to represent the TV coefficients of the intrinsic TV-IRF (${\left\{\Lambda_{j}\left(t_{k}\right)\right\}}_{j=0}^{j=n_{\lambda }}$); these basis functions were selected because they describe smoothly changing signals, such as the simulated TV parameters, very well. A total of 10 B-splines were used to represent each TV parameter since this was found to be the minimum order necessary to account for 99% of the variability of the true TV parameters. The B-splines knots were uniformly distributed along the cycle.

B-splines were also used to represent the TV, reflex static-nonlinearity (${\left\{\Gamma_{j}\left(t_{k}\right)\right\}}_{j=0}^{j=n_{\gamma }}$), and the numerator of the TV, reflex linear dynamics (${\left\{\Psi_{j}\left(t_{k}\right)\right\}}_{j=0}^{j=n_{\beta }}$). However, they could not be used to represent the parameters of the denominator due to technical limitations associated with the identification algorithm as described in Guarin and Kearney (2016). Consequently, Chebyshev polynomials of order 0–7 were selected as the basis functions to represent the coefficients in the denominator of the TV, reflex linear dynamics (${\left\{\Pi_{j}\left(t_{k}\right)\right\}}_{j=0}^{j=n_{\alpha }}$).

Chebyshev polynomials of order 0–4 were used to represent *TQ*_Δ_(*t*_*k*_), since we found that they provided a more parsimonious representation of the low-frequency component, *TQ*_Δ_(*t*_*k*_), than cubic B-splines.

Moreover, Chebyshev polynomials were used to parametrize the reflex, static-nonlinearity (${\left\{C_{j}\left({\overset{{}^{\circ}}{\theta }}_{p}\left(t_{k}\right)\right)\right\}}_{j=0}^{j=n_{c}}$). There are some advantages of using this polynomial representation: (i) the first-order polynomial is linear, $C_{1}\left({\overset{{}^{\circ}}{\theta }}_{p}\left(t_{k}\right)\right)={\overset{{}^{\circ}}{\theta }}_{p}\left(t_{k}\right)$, so that the estimated parameters can be used to validate whether a nonlinear model is needed or not; and (ii) the variance of the output is finite in its support, which guarantees the numerical stability of the estimation process. Polynomials of order 0–4 were used to approximate the TV static-nonlinearity.

As the gain of the Hammerstein system can be arbitrarily assigned to the static-nonlinearity or the linear dynamic element without affecting the output, we assigned the gain of the reflex pathway to the static-nonlinearity and fixed the gain of the linear dynamics to unity.

#### 3.1.4. Validation

The predictive ability of the estimated model parameters were quantified in terms of the Variance Accounted For (VAF) between the predicted and simulated torques. An average-VAF was computed for the entire simulation trial as described in step 5 of the identification algorithm. In addition, a TV-VAF was computed by dividing each gait cycle in 20 segments of equal length and computing the VAF between predicted and simulated signals for each segment.

The joint intrinsic static stiffness (*K*(*t*_*k*_)), viscosity (*B*(*t*_*k*_)) and inertia (*I*) were computed directly from the estimated TV-IRF by using a non-linear least-squares fit algorithm (Kearney et al., 1997), and compare to the simulated parameter.

The shape of the estimated reflex, TV, static-nonlinearity (which includes the reflex gain) was compared to that of the simulated TV, half-wave rectifier. The TV, reflex natural frequency (ω) and damping (ζ) were computed directly from the estimated, discrete-time parameters and compared to the simulated values.

##### 3.1.4.1. Ensemble identification algorithm

For comparison purposes we estimated the model parameters using the ensemble identification algorithm for estimation of the parallel-cascade model structure previously introduced by our group (Ludvig et al., 2011). This algorithm uses an ensemble only identification approach for estimating the TV parameters of the intrinsic and reflex dynamic joint stiffness.

##### 3.1.4.2. Time-invariant, dynamic joint stiffness model

Furthermore, a time-invariant (TI), dynamic joint stiffness model was estimated between the perturbation position and noisy torque signals using the entire record. The TI, intrinsic and reflex model parameters were estimated using the new algorithm with the orders of the basis functions used to represent the TV intrinsic and reflex model parameters set to one, forcing them to be a constant, all-ones vector.

### 3.2. Results

#### 3.2.1. Time-invariant results

**Figure 5:**
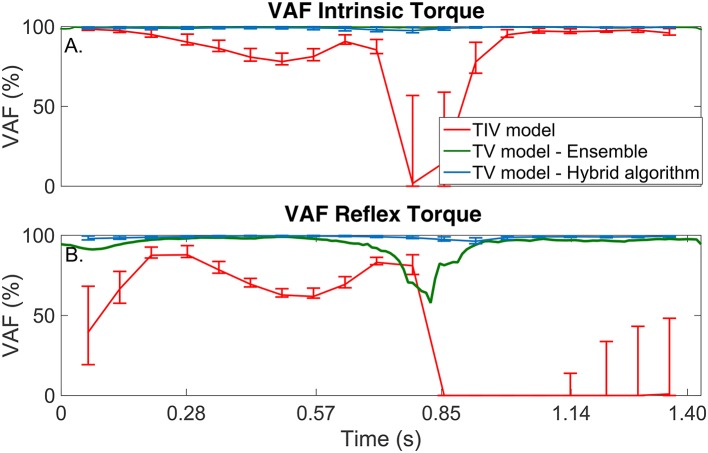
TV-VAF between the noise-free torque and that predicted by the time-invariant (red) and time-varying models, estimated with the ensemble (green) and hybrid (blue) algorithms. Bars represent the mean, 5th and 95th percentiles observed in 4,000 simulated cycles. **(A)** Intrinsic Torque, and **(B)** Reflex Torque.

The TI model did not predict the simulated torque well, the average-VAF was always less than 70% for both intrinsic and reflex torques. Furthermore, Figure 5 shows that the TV-VAF varied greatly across the cycle; it ranged between 0 and 99% for the intrinsic and between 0 and 90% for the reflex torque, indicating that the TI models did not captured the simulated system dynamics.

#### 3.2.2. Ensemble identification algorithm

The ensemble only identification algorithm required at least 400 input-output realizations to produced acceptable results. With this large data set, the average-VAF was larger than 90% for both intrinsic and reflex torques. Furthermore, Figure 5 shows that the TV-VAF for the intrinsic torque was greater than 98% at all points in the cycle, indicating that ensemble identification algorithm accurately captured the linear, intrinsic dynamics. However, the TV-VAF for the reflex models varied greatly across the cycle; it ranged between 55 and 99%, indicating that the ensemble identification algorithm did not captured the non-linear, reflex dynamics.

#### 3.2.3. Hybrid identification algorithm

The TV model predicted the output extremely well, the average-VAF was always larger than 99% for both intrinsic and reflex torques. Furthermore, Figure 5 shows that the TV-VAF for both the intrinsic and reflex torques was greater than 97% at all points in the cycle; the lowest values were observed around the portion of the cycle where the gain of the intrinsic and reflex pathways were smallest.

##### 3.2.3.1. TV intrinsic dynamic stiffness

**Figure 6:**
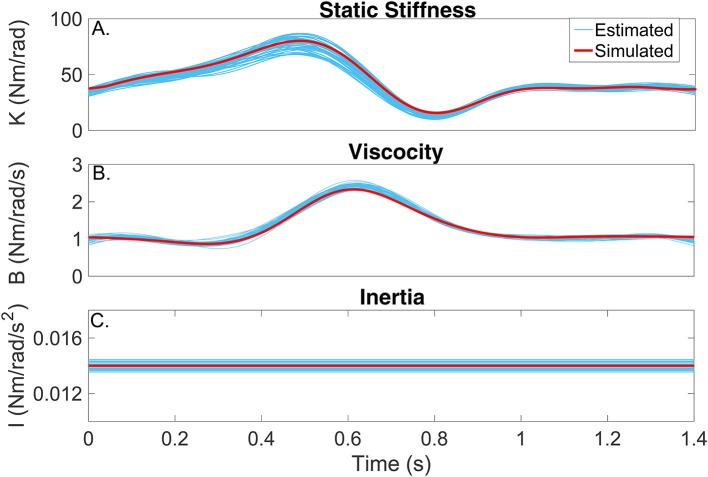
Intrinsic dynamic stiffness. Simulated (red) and estimated (blue) **(A)** static stiffness (*K*(*t*_*k*_)), **(B)** joint viscosity (*B*(*t*_*k*_)), and **(C)** limb's inertia (*I*(*t*_*k*_)) as a function of time.

Figures 6A–C compares the simulated (red) and estimated (blue) intrinsic static stiffness, viscosity and inertia, demonstrating that the estimated parameter tracked the true value very closely and with little variability in all 100 simulation trials.

##### 3.2.3.2. TV reflex dynamic stiffness

**Figure 7:**
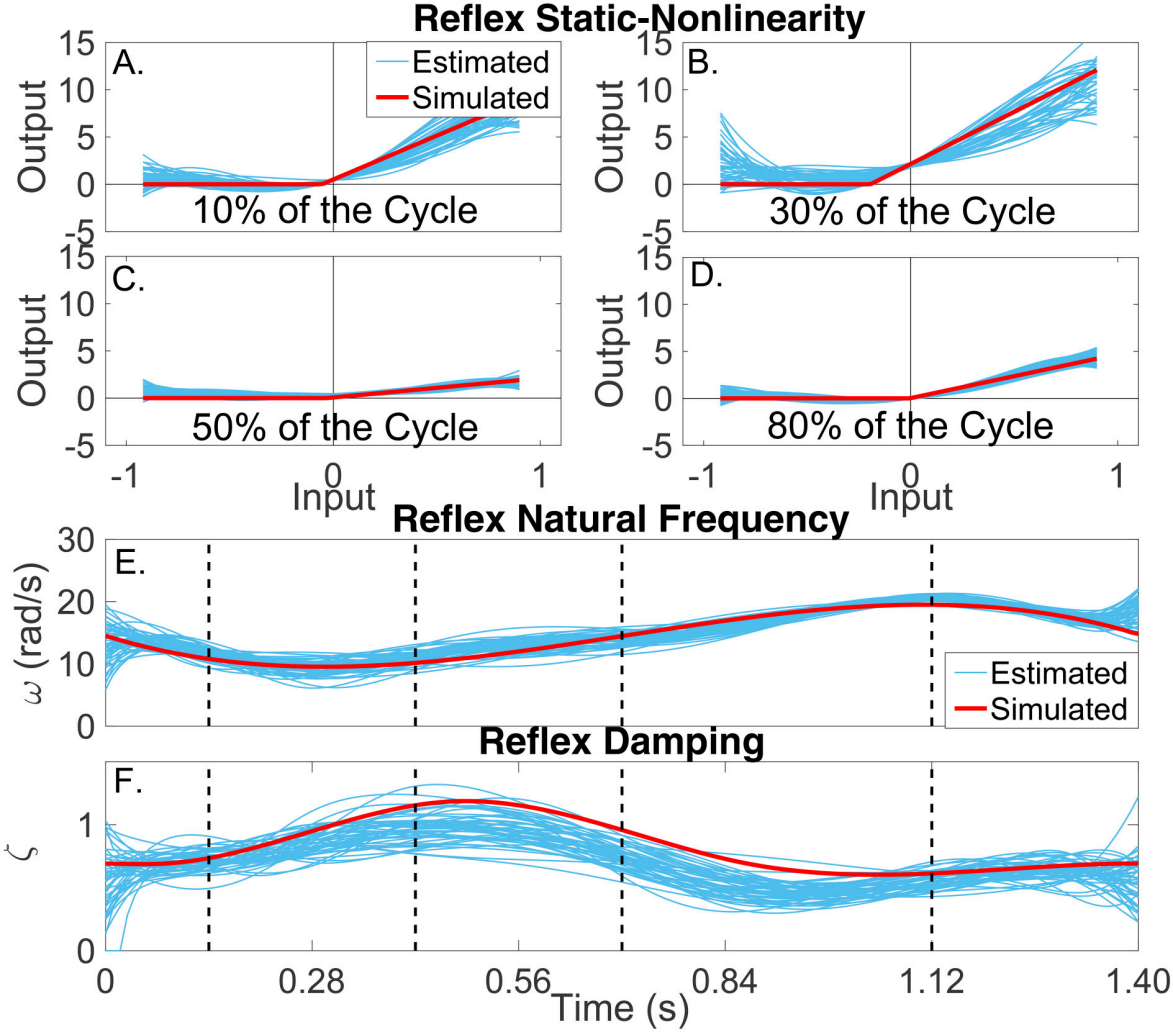
Reflex dynamic stiffness. **(A–D)** Snapshots of the the simulated (red) and estimated (blue) TV, static nonlinearity at the four points of the cycle indicated by vertical lines in **(D,E)**. Simulated (red) and estimated (blue) **(E)** reflex natural frequency (ω(*t*_*k*_)), and **(F)** damping (ζ(*t*_*k*_)) as a function of time.

Figures 7A–D present snapshots of the estimated and simulated TV static-nonlinearity at different points of the cycle. It is evident that the estimated, polynomial static-nonlinearity accurately tracked both the TV threshold and slope of the simulated half-wave rectifier. It can also be observed that the variability of the polynomial nonlinearity was smaller for velocities around zero. The bottom panels of the figure show the simulated and estimated natural frequency and damping of the reflex, linear dynamic element, demonstrating that the estimated parameters tracked the true values closely. The reflex damping was slightly underestimated; but this did no affect the VAF, indicating that the model output is less sensitive to the damping than to the other elements.

## 4. Experimental study

The practical utility of the new TV, identification algorithm was evaluated by using it to estimate the dynamic ankle stiffness from experimental data acquired during an imposed movement with constant voluntary torque. Data was acquired from one healthy subject who provided written informed consent. The experiment was approved by the McGill University Research Ethics Office.

### 4.1. Experimental methods

The subject lay supine with his left foot attached to the pedal of a stiff electrohydraulic actuator operating as a position servo, which prevented the subject from voluntarily moving its ankle, by means of a custom made fiberglass boot (Kearney et al., 1997). Ankle movement was restricted to dorsiflexion and plantarflexion, defined as positive and negative angles respectively with respected to a zero-position reference, taken 90° between the foot and shank.

Ankle position, torque, and surface EMG from the medial and lateral gastrocnemius (GM and GL), soleus (SOL) and tibialis anterior (TA) were measured, filtered at 400 Hz to prevent aliasing and sampled at 1 kHz by a 16-bit A/D converter. Data were low-pass filtered and decimated to 100 Hz for analysis. Surface EMG electrodes were placed following the SENIAM recommendations (Hermens et al., 2000).

During each experimental trial the actuator moved the ankle to zero position and held it there for a 1 min. Then, an unperturbed trajectory, consisting of the angle of the ankle joint during walking, with a duration of 2 s, was applied; this trajectory was extracted from Lee and Hogan (2015). The trajectory was repeated periodically 30 times; the trial was repeated twice to obtain a total of 60 cycles.

The unperturbed trials were performed two times. The first time the subject was instructed to (i) be relaxed, and (ii) not react to the imposed movement. The second time, the subject was instructed to: (i) maintain a constant plantarflexion torque corresponding to 10% of its maximum torque at zero position (recorded previously at 70 Nm); and (ii) not react to the imposed movement. To assist with this task, the subject was presented with a visual feedback of a low-pass filtered (0.7 Hz) version of the measured torque minus the passive torque recorded in the previous experiment. The subject was allowed to train for several minutes before the beginning of the trial.

The trials were then repeated using a perturbed ankle trajectory by adding a PRALDS signal, similar to that used in the simulation study, to the walking trajectory. In the perturbed trials the subject was instructed to: (i) maintain a constant plantarflexion torque corresponding to 10% of his maximum torque; and (ii) not react to the imposed movement and perturbations.

Perturbed and unperturbed position and torque records were subtracted to give the perturbation position (θ_*p*_(*t*_*k*_)) and torque (*TQ*_*p*_(*t*_*k*_)). Each trial was then divided into identification and validation segments; 40 cycles were used for parameters estimation and the remaining 20 cycles for model validation; validation data was not used for parameter estimation, only for model validation. The model was validated by computing the average-VAF between the measured and predicted torques for the validation data.

The identification procedure was started with the same number of basis functions used in simulations; a subset of basis functions was then selected automatically by using a sparse identification algorithm, which forces the weights associated to basis function that do not contribute to the reduction of the prediction error to zero so that they can be discarded Guarin and Kearney (Submitted).

Finally, joint velocity was computed by numerically differentiating the perturbation position signal. Then, the reflex delay was computed by finding the time difference between the positive peaks in the joint velocity signal and the corresponding peaks in the soleus EMG signals associated with the reflex response.

### 4.2. Results

#### 4.2.1. Typical trial

**Figure 8:**
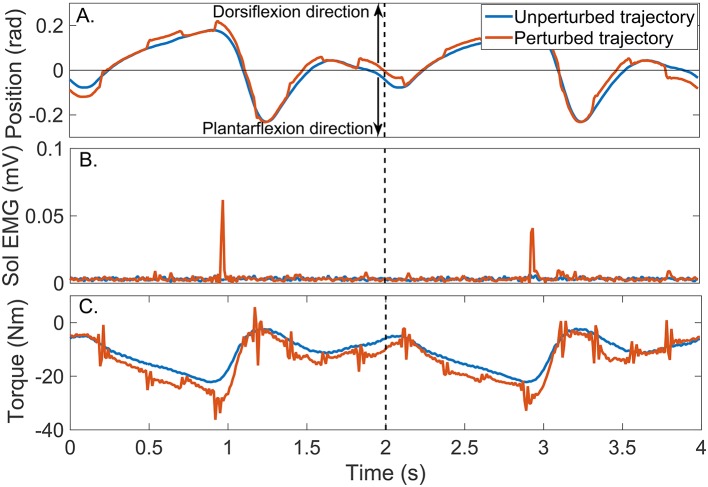
Typical signals recorded during a un-perturbed (blue) and perturbed (brown) trajectory. **(A)** position, **(B)** Soleus EMG, and **(C)** torque.

**Figure 9:**
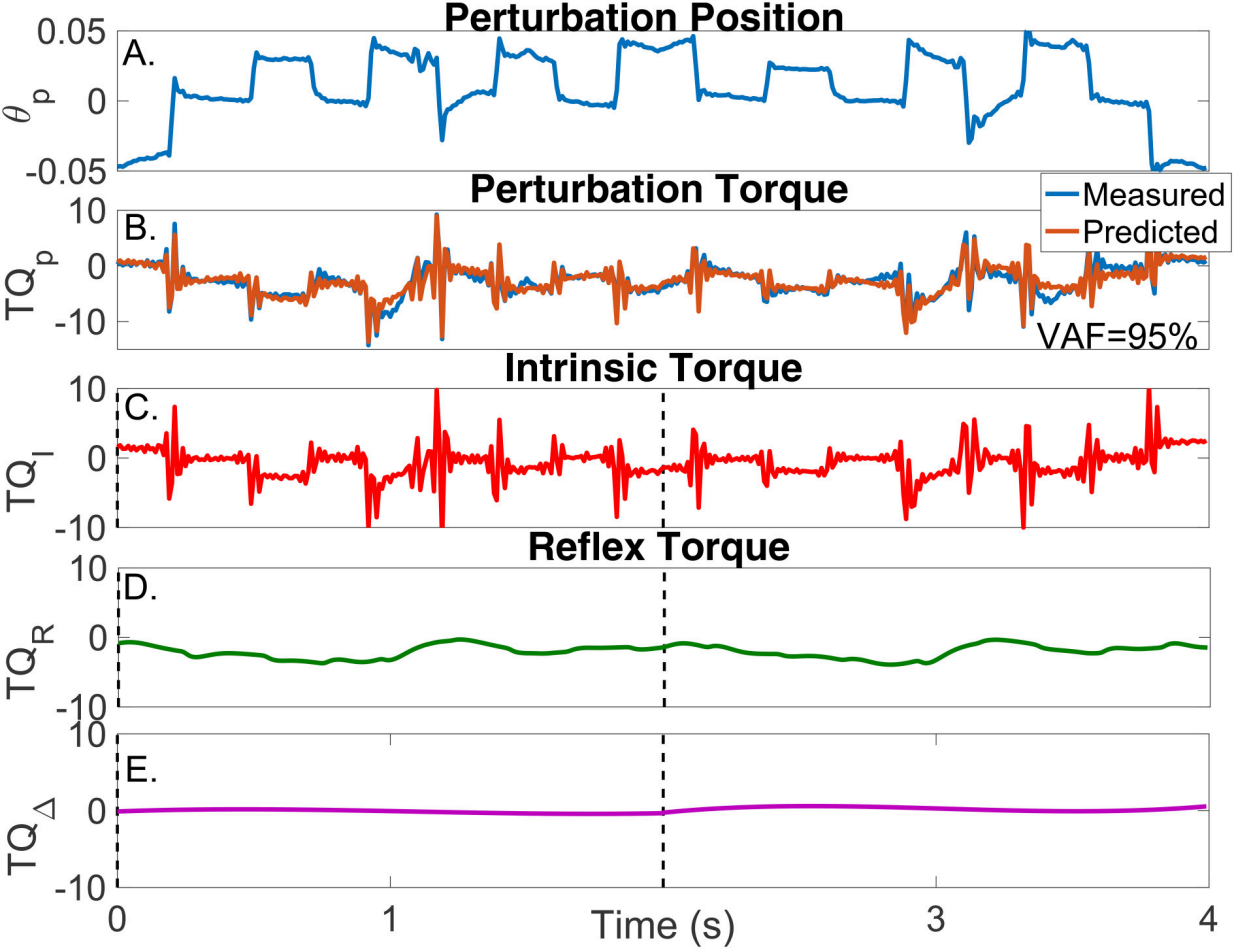
Results for a typical validation trial as a function of time. **(A)** Perturbation position input, **(B)** Measured (blue) and predicted (brown) perturbation torque, **(C)** Estimated intrinsic torque, **(D)** Estimated reflex torque and **(E)** Estimated additional torque. The predicted perturbation torque is the sum of the intrinsic, reflex and additional torques.

Figure 8 shows two cycles of the perturbed and unperturbed position, where the dorsiflexion and plantarflexion directions are indicated with black arrows; soleus EMG, and torque records. The blue lines in Figures 9A,B show the corresponding perturbation position (θ_*p*_(*t*_*k*_)) and torque (*TQ*_*p*_(*t*_*k*_)).

#### 4.2.2. Time-invariant results

The output of the TI model estimated from these data did not predict the ankle torque very well (data not shown). The average-VAF never exceeded 75%, demonstrating that a TV model is required to capture the system dynamics.

#### 4.2.3. Time-varying model

In contrast, the estimated TV model predicted the measured torque very well; the average-VAF for the validation trials was never less than 95%. The brown line in Figure 9B shows the predicted total torque (the sum of intrinsic, reflex and additional torques) for two validation trials, whose average-VAF was 95%. This excellent agreement between measured and predicted torques indicates that the TV model estimates accurately captured the system dynamics. Figures 9C–E also show the predicted ${\hat{TQ}}_{I}\left(t_{k}\right)$, ${\hat{TQ}}_{R}\left(t_{k}\right)$, and ${\hat{TQ}}_{\Delta }\left(t_{k}\right)$ as a function of time. The intrinsic torque accounted for most of the variance of the measured data for this experiment; however, both the reflex torque and *TQ*_Δ_(*t*_*k*_) were non-zero for all the cycle.

##### 4.2.3.1. TV intrinsic dynamic stiffness

**Figure 10:**
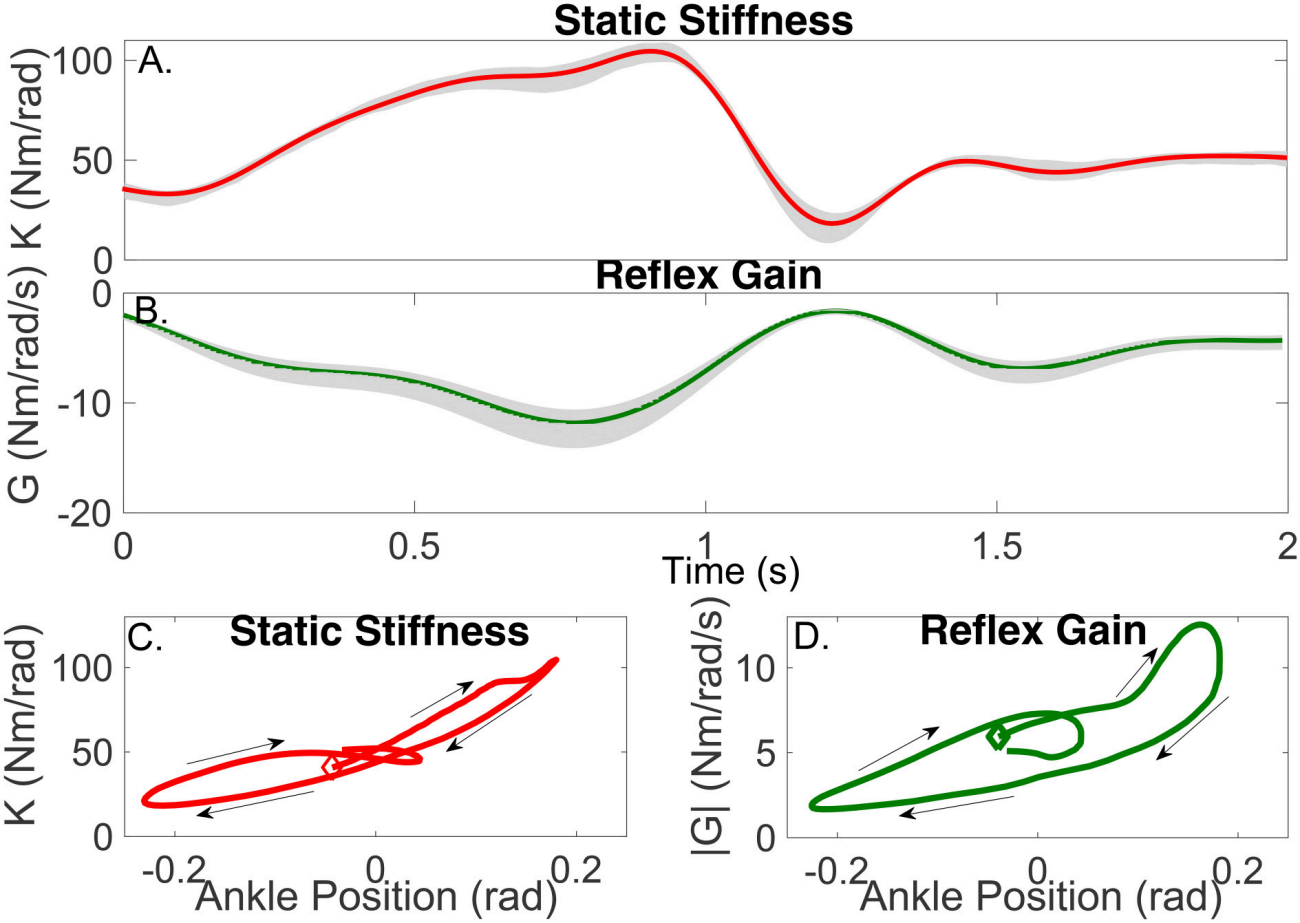
Gain of intrinsic and reflex stiffness as a function of time **(A,B)** and ankle position **(C,D)**. Shadows represent the 95% confidence interval. The beginning of the cycle is indicated by the diamond, arrows show the progression of the cycle.

The parameters of the TV-IRF describing the intrinsic dynamic stiffness underwent large, fast changes throughout the cycle. Figure 10A presents the variation in the intrinsic static stiffness along with the 95% confidence interval, computed by a bootstrap analysis with 100 repetitions (Press, 2007). The intrinsic elasticity increased three fold (from 35 Nm/rad to 100 Nm/rad) in the first half of the cycle, it then decreased sharply and stayed nearly constant during the remainder of the cycle.

Figure 10C shows the static intrinsic stiffness as a function of ankle position, demonstrating that: (i) static intrinsic stiffness is larger in plantarflexion than dorsiflexion; (ii) the relation between joint position and intrinsic elasticity is nonlinear and is influenced by the immediate history of the movement, as different values of the static stiffness were observed for the same joint position during different parts of the cycle.

**Figure 11:**
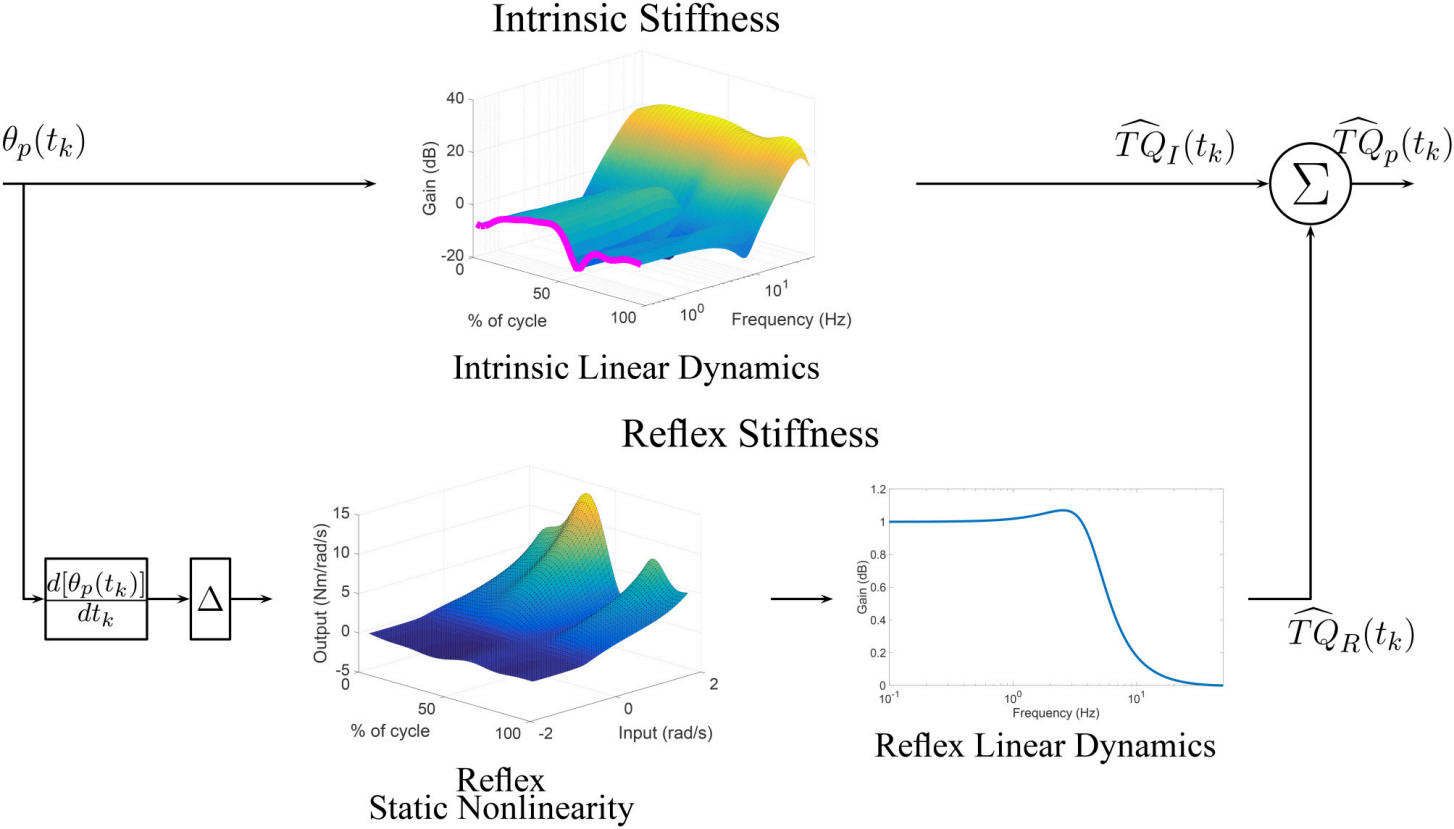
TV, Parallel-Cascade model estimated from experimental data. Intrinsic dynamic stiffness modeled as a TV-IRF model. Reflex dynamic stiffness modeled as a Hammerstein system with TV static-nonlinearity followed by a time-invariant, low-pass filter.

Furthermore, the upper pathway of Figure 11 shows the time-frequency response of the TV-IRF as a function of the cycle with the purple line indicating the static stiffness. The intrinsic dynamic stiffness showed a high-pass behavior, typically observed during stationary experiments (Kearney et al., 1999), and underwent large, fast changes in the low and mid-frequency components, related to the joint visco-elastic properties, throughout the gait cycle. The high-frequency components, related to the joint inertial properties, did not change much throughout the cycle.

Attempts to fit a second order model to the estimated TV-IRF provided inaccurate parametric models unable to properly describe the intrinsic joint dynamics. This is consistent with recent evidence that joint mechanical properties are more complex than second order (Sobhani Tehrani et al., 2017).

##### 4.2.3.2. TV reflex dynamic stiffness

The parameters of the polynomial nonlinearity representing the reflex, static-nonlinearity underwent large, fast changes throughout the gait cycle. Figure 10B shows the variation in the reflex gain, computed as the slope of the static nonlinearity, along with the 95% confidence interval. The reflex gain increased six fold (from -2 Nm/rad/s to -12 Nm/rad/s) during the first half of the cycle, and then decreased rapidly to an almost constant value for the remainder of the cycle.

Figure 10D shows the reflex gain as a function of ankle position. This plot indicates that: (i) the reflex gain is larger in plantarflexion than dorsiflexion; (ii) the relation between joint position and reflex gain is nonlinear and is influenced by the immediate history of the movement.

The parameters of the second-order, linear system representing the reflex, linear dynamics did not vary much. The lower pathway of Figure 11 summaries the TV reflex behavior. It shows the TV, static-nonlinearity as a function of cycle and the frequency response of the linear dynamics. The estimated static-nonlinearity resembles a half-wave rectifier; whose gain underwent large, fast changes throughout the cycle. The linear dynamics are low-pass in nature and did not vary throughout the cycle. The shape of the static nonlinearity and the cut-off frequency of the linear dynamic element are similar to what has been observed in stationary experiments (Kearney et al., 1999).

## 5. Discussion and conclusions

This paper presents a new model parameterization and identification algorithm for the accurate estimation of the intrinsic and stretch reflex components of dynamic joint stiffness during movement. The algorithm combines ensemble and deterministic approaches to estimate TV model parameters from position and torque records. Simulations demonstrated that the new algorithm successfully decomposed the dynamic joint stiffness into its intrinsic and reflex components, and accurately tracked the fast, large changes in the parameters of each pathway using only 40 cycles in the presence of complex, experimental noise. This represents a much-needed improvement over ensemble only algorithms, which were not able to accurately track the changes in intrinsic and reflex dynamics even after using 400 cycles. Furthermore, the practical application of the method was successfully demonstrated by using it to track the changes in ankle stiffness in a human subject in an experiment that involved an imposed walking movement with constant muscle activation. The excellent agreement between the predicted and experimental torques demonstrated that the new methodology accurately describes the modulation of dynamic ankle stiffness during the movement.

### 5.1. Methodological issues and limitations

Methods that estimate TV, dynamic joint stiffness make three underlying assumptions: (i) The small perturbations applied to the joint do not change much the operating point (Kearney et al., 1997); (ii) the mechanical response of the joint to small perturbations and to large changes in the operating point are linearly superimposed (Gottlieb and Agarwal, 1978); and (iii) changes in the system dynamics with joint position and torque can be described by a set of local models at each point in time (Bennett et al., 1992). The excellent agreement between the predicted and measured torques suggests that these assumptions hold for the slow ankle trajectory used in our experiments, which resembles slow walking. However, it remains to be determined if these assumptions are valid during faster joint movements.

Our methodology leverages these assumptions and introduces a novel parameterization of the parallel-cascade model where the time-course of the local models parameters are approximated by a linear combination of basis functions. These approximations transform the TV model into a set of TI models at the cost of increasing the number of free parameters. This raises a number of issues with the new algorithm:

First, the number of free-parameters increased by the re-parameterization procedure; therefore, the identification algorithm requires large data sets for accurate parameter estimation. This limitation was addressed here by combining the basis function expansion with an ensemble identification approach, which uses multiple, input-output trials with the same TV behavior. However, compared with ensemble-only identification methods, our algorithm requires a lot less repetitions, which translates into much shorter experiments making it easier to acquire enough trials with the same TV behavior.

Second, the type and number of basis functions used to parametrize the TV coefficients must be known *a priori*; the quality of the parameter estimates will depend on selecting a set of basis functions capable of efficiently describing the TV parameter changes. This study uses B-splines and Chebyshev polynomials, both of these basis functions are well suited to describe smooth parameter changes. B-splines are useful when the changes are rapid, polynomial basis are adequate to approximate low-frequency trends (Zou et al., 2003; He et al., 2013).

Third, this method was designed to work with data measured in open-loop. This is the case in experiments, such as ours, where a very stiff actuator, acting as a position servo, imposes a desired joint trajectory so that any torques produced by the joint in response to the perturbation do not result in position changes. That is, the relation between joint position and torque is open-loop. In contrast, during most natural movements, the joint interacts with a compliant load so that torques generated in response to position changes will in turn modify the joint position, resulting in closed-loop measurement of joint position and torque. Most methods for identification of dynamic join stiffness have been designed to work with open-loop data, and using these methods with data measured in closed-loop will lead to biased parameter estimates (Kearney and Hunter, 1990).

The method presented is an open-loop method; however, it can be reformulated to work with data measured in closed-loop. This would require adopting a new model of dynamic joint stiffness, with the feedforward and feedback pathways comprising the intrinsic and reflex components respectively (Van der Helm et al., 2002). The algorithm described here for identification of intrinsic dynamics cannot be used with closed-loop data as it will provide biased results. However, an instrumental variable algorithm for parameter identification can be used directly to estimate the intrinsic component from closed-loop data as described in Guarin and Kearney (2016). The method presented here for estimation of reflex dynamic stiffness uses instrumental variables and so can be applied directly to estimate the nonlinear, Hammerstein system representing the reflex dynamics from data measured in closed-loop (Young, 2011).

Moreover, our implementation of the identification algorithm assumes that the time-varying behavior is periodic, so that the initial conditions of each trial in the ensemble will be the same facilitating their estimation. However, the algorithm could be modified to work with non-periodic data; this would require estimating the initial conditions of each trial in the ensemble as part of the identification problem as done in Jalaleddini et al. (2017).

Finally, the algorithm relies on knowledge of the reflex response delay to accurately separate the intrinsic and reflex components from the measured position and torque data. It assumes that the delay remains constant throughout the cycle. We measured the reflex delay from joint velocity and soleus EMG signals, and found that it remained constant across the cycle.

### 5.2. Simulation study

System identification methods are often validated using idealistic input and noise sequences. However, the performance of these algorithms often degrades when applied to experimental data, where inputs are non-ideal and the noise is neither zero-mean, nor white. Our simulation was intended to mimic real experiments; model parameters were based on those reported in the literature; inputs signals had limited bandwidth; and the noise was extracted from experimental observations. Consequently, we believe that our simulation results are more relevant to experimental conditions.

As Figures 6,7 show, the simulated, intrinsic and reflex stiffness model parameters were accurately estimated by the identification algorithm. The large variability in the polynomial nonlinearity at large velocities is likely related to the amplitude distribution of the velocity signal, which despite having velocities distributed over the entire sets of values, is highly concentrated around zero (Jalaleddini and Kearney, 2013). Finally, the reflex natural frequency was accurately estimated, and the reflex damping was slightly underestimated. However, this did not affect the prediction ability of the estimated models, indicating that the model output is not very sensitive these small differences in the damping.

### 5.3. Experimental study

We also applied the new method to actual experimental data to estimate the intrinsic and reflex dynamic ankle stiffness during an movement. Results showed that the model structure predicted the output torque to novel perturbation sequences, indicating that the estimated model successfully captured the TV, nonlinear dynamics.

Figure 10A shows that the static stiffness changed dramatically during the imposed movement, it increased substantially during the first part of the cycle (from around 35 Nm/rad to 100 Nm/rad) and then sharply decreased (to 20 Nm/rad) in just 200 ms, it maintained a nearly constant value for the remainder of the cycle. Figure 10C demonstrates that the ankle static stiffness can take different values for the same ankle angle depending on the immediate history of the movement. This demonstrates a true TV behavior in the joint neuromuscular properties, and not just a static-nonlinear dependency on joint position, as has been previously assumed (Sobhani Tehrani et al., 2013; Jalaleddini et al., 2015).

Figure 10B shows there were also large TV changes in the reflex gain, it increased (from around −2 Nm/rad/s to −12 Nm/rad/s) during the first 800 ms of the cycle, then rapidly decreased to almost zero over the next 400 ms, and then remained relatively constant for the remainder of the cycle. Moreover, Figure 10D demonstrated that there is a significant history-dependent behavior, with the reflex gain showing values with a difference of up to 300% for the same value of joint position. The other components of the reflex pathway did change during the movement.

Furthermore, the reflex gain attained its maximum value at least 100 ms before the intrinsic static stiffness, this is consistent with the idea that the tonic stretch reflex might mediate the changes in muscle activation, leading to an increased intrinsic static stiffness Feldman and Levin (2009). In addition, the history-dependent behavior was observed in both the intrinsic static stiffness and reflex gain; however, as Figure 10D demonstrates, this behavior was much more significant for the reflex than the intrinsic component. This might be explained by the fact that reflex dynamic stiffness is generated only by the active muscle response to stretch activation whereas intrinsic dynamic stiffness is generated by both active and passive components.

We conclude that the new algorithm will be a useful tool in the study of dynamic joint stiffness during TV conditions and that it will help further the understanding of the modulation of this system during function.

## Author contributions

All authors listed, have made substantial, direct and intellectual contribution to the work, and approved it for publication.

### Conflict of interest statement

The authors declare that the research was conducted in the absence of any commercial or financial relationships that could be construed as a potential conflict of interest.

## Footnotes

^1^https://github.com/dguari1/Frontiers2017.
